# The impact of gun violence restraining order laws in the U.S. and firearm suicide among older adults: a longitudinal state-level analysis, 2012–2016

**DOI:** 10.1186/s12889-020-08462-6

**Published:** 2020-04-07

**Authors:** Altaf Saadi, Kristen R. Choi, Sae Takada, Fred J. Zimmerman

**Affiliations:** 1 Massachusetts General Hospital, Harvard Medical School, Boston, MA 02114 USA; 2 University of California Los Angeles School of Nursing, Los Angeles, CA 90024 USA; 3 Division of General Internal Medicine & Health Services Research, David Geffen School of Medicine, University of California Los Angeles, Los Angeles, CA 90024 USA; 4 Department of Health Policy and Management, Fielding School of Public Health University of California, Los Angeles, 90024 USA

**Keywords:** Older adults, Suicide, Gun policy

## Abstract

**Background:**

Older adults complete suicide at a disproportionately higher rate compared to the general population, with firearms the most common means of suicide. State gun laws may be a policy remedy. Less is known about Gun Violence Restricting Order (GVRO) laws, which allow for removal of firearms from people deemed to be a danger to themselves or others, and their effects on suicide rates among older adults. The purpose of this study was to examine the association of state firearm laws with the incidence of firearm, non-firearm-related, and total suicide among older adults, with a focus on GVRO laws.

**Methods:**

This is a longitudinal study of US states using data from 2012 to 2016. The outcome variables were firearm, non-firearm and total suicide rates among older adults. Predictor variables were [1] total number of gun laws to assess for impact of overall firearm legislation at the state level, and [2] GVRO laws.

**Results:**

The total number of firearm laws, as well as GVRO laws, were negatively associated with firearm-related suicide rate among older adults ages 55–64 and > 65 years-old (*p* < 0.001). There was a small but significant positive association of total number of firearm laws to non-firearm-related suicide rates and a negative association with total suicide rate. GVRO laws were not significantly associated with non-firearm-related suicide and were negatively associated with total suicide rate.

**Conclusion:**

Stricter firearm legislation, as well as GVRO laws, are protective against firearm-relate suicides among older adults.

## Background

Access to firearms poses potential public health dangers for older adults with regards to attempted or completed suicides, particularly as older adults complete suicide at a disproportionately higher rate compared to the general population [1, 2]. One study identified the firearm suicide rate for people age 65 and older in the United States to be 11.8 per 100,000 [3], more than three times higher compared to that in other high-income countries [3]. Suicide has been shown to be often an impulsive act [4], and suicide rates are higher where circumstances make suicide more easily visible and attainable [5]. Readily available firearms are one way in which the means of suicide is both more visible and more attainable. In fact, both regional and state-level analyses of household firearm ownership has been shown to be associated with increased suicides, including among older adults [6–9]. Firearm suicide is of particular concern among older adults who may be experiencing cognitive decline and dementia that might predispose patients to depression, confusion, agitation, aggression, or paranoia. In one literature review of suicidal behavior in people with dementia, suicidal behavior was deemed more common earlier in the dementia course when there remains awareness and insight into impending cognitive decline [10]. Studies have found the range of persons with dementia living in a household with a firearm to be from 18 to 60% [11–13], including one study that found nearly a quarter keep at least one gun unlocked and loaded [13].

Efforts to reduce the risk of impulsive suicides by firearms have included household- and policy-level strategies such as safe gun storage and legislation that restricts firearm access. Policies restricting firearm ownership have been associated with a decline in firearm suicide rates in many countries [4], including the United States [14, 15]. The impact on non-firearm suicides is less clear. In one study examining the impact of state gun laws on both firearm and non-firearm suicides, there was a reduction in firearm suicides but not in non-firearm suicides in the population as a whole [16]. Among older individuals, however, there was an increase in non-firearm suicides seen [16], raising concerns about whether additional policies are needed for subgroups who may shift to non-firearm suicides. Of note, while non-firearm means of suicide are still impulsive, they are less likely to be lethal when compared to firearms (e.g. drug ingestion). Fewer studies have looked at the impact of specific subtypes of firearm laws on suicide, although universal background checks and violent misdemeanor laws have not been shown to be related to overall suicide rates [17].

In the aftermath of several high-profile mass shootings, including that at Marjory Stoneman Douglas High School shooting in Florida that garnered national protests against legislative inaction on gun violence, several states have introduced Gun Violence Restraining Order (GVRO) laws. GVRO laws—also called extreme risk protection orders or more generically “red flag” laws—allow law enforcement and/or families to petition a judge for removal of firearms from someone who may be a danger to themselves or others (e.g. revealing “red flags”). Since the Parkland shooting, 20 states have proposed GVRO legislation [18]. Previously, only five states—Connecticut, Indiana, California, Oregon and Washington—had implemented some version of a GVRO into law. Although not instituted with older adults in mind, these laws have been postulated to address the issue of gun ownership and suicide risk among older adults [19]. But research on the impact of GVRO laws on suicides among older adults remains sparse, with existing studies focusing on population effects across age groups [20, 21].

This study tests the association of state firearm laws with the incidence of firearm- and non-firearm-related suicide among older adults, with a particular focus on GVRO laws that allow for removal of firearms from people deemed to be a danger to themselves or others. We hypothesize that, relative to states with fewer firearm laws, states with higher number of firearm-related laws will 1) have lower firearm-related suicides; 2) higher non-firearm-related suicides due to a potential shift from one means of suicide to another; and 3) fewer overall suicides. We hypothesize that GVRO laws will be associated with fewer firearm-related and overall suicides.

## Methods

### Study design and data source

This study used state-level data to conduct a longitudinal analysis as we have done previously [22]. The study was determined to be exempt from Institutional Review Board regulation at the University of California, Los Angeles because it uses de-identified, publicly available, state-level data.

### Outcome variable

The primary outcome was firearm, non-firearm-related, and total suicide rates among older adults obtained from the Centers for Disease Control Web-based Injury Statistics Query and Reporting System (WISQARS) from 2012 to 2016 [23]. We selected 2012 to 2016 as a timeframe because this time period encompassed Medicaid expansion under the Affordable Care Act and our analysis used Medicaid generosity as a covariate. Rates for older adults were studied in two age categories, ages 55 to 64 years and ages 65 and older. The 55–64 age category was included to encompass an age group more likely to be diagnosed with early-onset dementias, which may predispose more to impulsive acts and have an earlier average age of onset than other dementia types (e.g. frontotemporal dementia) [24].

### Exposure variables

The primary exposure variables were (1) total number of gun laws to assess for impact of overall stricter firearm legislation at the state level, and (2) Gun Violence Restraining Order (GVRO) laws. Each U.S. state can legally and independently regulate firearms, thereby having a different number of gun laws. These variables were obtained from the State Firearm Laws Database, which compiles data on state firearm policy from 1991 to 2016 on 133 firearm laws in 14 categories [25]. There were two types of GVRO laws, one that allows for a family member or law enforcement officer to initiate a procedure to confiscate firearms from any person who represents a threat to themselves or others, and another that permits only law enforcement officers to initiate this process. States were dichotomized as either having or not having GVRO laws.

### Covariates

We used the following state-level data to adjust for characteristics associated with firearm-related suicide in prior research studies: poverty rate [26, 27], Medicaid generosity (percentage of the U.S. Federal Poverty Line that qualifies a parent for Medicaid benefits based on income per state) [28, 29], average state population density weighted by county (sum of the average density for all counties in a given state, for each year: [county population / county land area] * [county population / state population]) [30, 31], sex rate (percentage of the state population that is female) [32], and rate of older adults (percentage of the state population that is ages 55 to 64 and 65 and older) [32]. Some of these variables were particularly important among a population of older adults. For example, suicide rates are higher among older male adults than female adults [3].

### Data analysis

We used fixed effects linear regression models to explore the relationships between exposure variables and outcomes, adjusting for the covariates listed above. Models were also adjusted for year to account for secular trends. We first estimated models for firearm-related suicide rate among older adults in the two age categories (ages 55–64, and > 65 years-old) to understand whether there was a protective association to total number of firearm laws. Then, we repeated the models predicting non-firearm-related suicide rates among both groups and total suicide rate (all age groups, any means) to allow for comparison of the role of total number of firearm laws in firearm-related versus non-firearm-related suicide rates. These models were then re-estimated with an indicator for the presence of at least one GVRO law as the main predictor rather than total number of firearm laws (the total number of firearm laws variable was not included in these models).

## Results

From 2012 through 2016, the average percentage of firearm-related suicide among adults ages 55 to 64 was 12.1, and 12.8% among adults ages 65 and over. For states whose population comprised greater than 50% females, the percentage of suicides was lower in both age groups (Ages 55–64: 11.4% for majority female states versus 14.5% for equal gender distribution or majority male states; Ages 65+: 12.2% for majority female states versus 15.6% for equal gender distribution or majority male states). Across state-years (i.e. observations) in the sample, the count of state gun laws ranged from a low of 3 laws (Vermont 2012) to a high of 104 laws (California 2016). There were 12 state-years with at least one GVRO law in place among total of 4 states (California, Washington, Indiana, Connecticut). We summarize the study variables in Table 1.

**Table 1:** Summary of Study Variables

Variable	Mean	SD
Poverty rate (%)	17.88	4.05
Medicaid generosity (% of FPL)	0.94	0.46
Population density (per 1000 county population)	1.11	2.37
Number of firearm laws	26.16	25.3
Older adult (age > 65) population share (%)	13.26	1.11
Female population share (%)	51.00	0.84

**Footnotes**. This table provides summary statistics for analytic variables over the entire study period (2012–2016) among US states (*N* = 242). *FPL* Federal Poverty Line. There were 12 states (5% of

In unadjusted analysis, each additional firearm law was associated with a significant 0.13% decrease in firearm-related suicide among older adults in both age categories (Ages 55–64: SE = 0.01, *P* < .001; Ages 65+: SE = 0.01, *P* < .001). Table 2 shows the results of the fully adjusted models. With adjustments for sociodemographic covariates, total number of firearm laws remained negatively associated with firearm-related suicide among both age groups, with each additional firearm law associated with a 0.1% decrease in firearm-related suicide (*p* < 0.001). There was a small but significant positive association of total number of firearm laws to non-firearm-related suicide for both age groups (for both age groups: 0.02% increase, *P* < .001). There was a negative association between number of firearm law and total suicide for both age groups (Ages 55–64: β = − 0.07; SE = 0.01; *P* < .001; Ages 65+: β = − 0.06; SE = 0.01; *P* < .001) (Appendix 1).

**Table 2:** Relationship Between Total Number of Firearm Laws and Suicide Rate Among Older Adults from 2012 to 2016 in the US

Older Adults (> 65 years)	Firearm-related suicide rate	Non-firearm related suicide rate
	Model 1 (*R*^*2*^ = 0.58)	Model 2 (*R*^*2*^ = 0.38)
	β(SE)	β(SE)
Number of firearm laws	−0.10 (0.01)^b^	0.02 (0.01)^b^
Poverty rate	0.37(0.06)^b^	−0.01(0.03)
Population density	- < 0.01(<.01)^b^	<.01(<.01)
Medicaid generosity	0.11(0.54)	0.18(0.26)
Older adult population share	0.35(14.60)	28.49(7.14)^b^
Female population share	− 158.00(38.03)^b^	−143.00(20.10)^b^
Older Adults (55–64 years)	Firearm-related suicide rate	Non-firearm related suicide rate
	Model 4 (*R*^*2*^ = 0.64)	Model 5 (*R*^*2*^ = 0.34)
	β(SE)	β(SE)
Number of firearm laws	−0.10(0.01)^b^	0.02(0.01)^b^
Poverty rate	0.34(0.05)^b^	−0.05(0.03)
Population density	- < 0.01(<.01)^b^	<.01(<.01)
Medicaid generosity	0.02(0.43)	0.531 (0.27)^a^
Older adult population share	−8.76(17.83)	−6.54(11.06)
Female population share	−111.90(25.68)^b^	− 119.10(17.71)^b^

**Footnotes**. SE = standard error; *N* = 242. This table displays fixed effects models of state-level sociodemographic and policy factors predicting firearm- and non-firearm-related suicide rates among older adult populations in the US, excluding the District of Columbia and US territories. Models are adjusted for year. ^a^Value is significant at the 0.05 level. ^b^Value is significant at the 0.01 level

For the unadjusted GVRO models, we found that the presence of a GVRO law was associated with a 3.9% decrease in firearm-related suicide among older adults ages 65+ (*P* < .001) and a 3.8% decrease among older adults ages 55–64 (*P* < .001). After adjustments, GVRO laws remained associated with a 2.5% decrease in firearm-related suicide among older adults ages 65+ (*P* < .001) and a 2.4% decrease among older adults ages 55–64 (*P* < .001) (Table 3). GVRO laws were not significantly associated with non-firearm-related suicide for either age group. The association between the presence of a GVRO law and total suicide for both age groups was negative (Ages 55–64: β = − 2.49; SE = 1.12; *P* < .05; Ages 65+: β = − 2.55; SE = 1.10; *P* < .05) (Appendix 1).

**Table 3:** Relationship between GVRO Laws and Suicide Rate Among Older Adults from 2012 to 2016 in the US

Older Adults (> 65 years)	Firearm-related suicide rate	Non-firearm related suicide rate
	Model 7 (*R*^*2*^ = 0.47)	Model 8 (*R*^*2*^ = 0.33)
	β(SE)	β(SE)
GVRO law	−2.54(1.13)^a^	−0.35(0.48)
Poverty rate	0.49(0.06)^b^	−0.05(0.03)
Population density	- < 0.01(<.01)^b^	<.01(<.01)^a^
Medicaid generosity	−1.18(0.54)^b^	0.71(0.24)^b^
Older adult population share	19.05(16.10)	23.88(7.35)^b^
Female population share	−240.80(40.72)^b^	−118.70 (20.02)^b^
Older Adults (55–64 years)	Firearm-related suicide rate	Non-firearm related suicide rate
	Model 10 (*R*^*2*^ = 0.51)	Model 11 (*R*^*2*^ = 0.30)
	β(SE)	β(SE)
GVRO law	−2.45(0.97)^a^	−0.02(0.53)
Poverty rate	0.48(0.06)^b^	−0.09(0.03)^b^
Population density	- < 0.01(<.01)^b^	<.01(<.01)
Medicaid generosity	−1.81(0.44)^b^	0.96(0.24)^b^
Older adult population share	35.42(19.95)	−17.10(10.90)
Female population share	−197.20(27.83)^b^	−99.66(17.21)^b^

**Footnotes**. *GVRO* Gun violence restraining order; *SE* Standard error; *N* = 242. This table displays fixed effects models of state-level sociodemographic and policy factors predicting firearm- and non-firearm-related suicide rates among older adult populations in the US, excluding the District of Columbia and US territories. Models are adjusted for year. ^a^Value is significant at the 0.05 level. ^b^Value is significant at the 0.01 level

## Discussion

This study supports the hypothesis that states with a higher number of firearm-related laws had lower percentages of firearm-related suicides among older adults compared with states with fewer firearm laws, building on previous work that has found this same relationship in the general population [14]. However, there appeared to be a slight increase in non-firearm-related suicides in this age group, which was also seen in a recent study by Ghiani et al. (2019), although they observed no impact on state firearm-related laws on non-firearm suicides overall [16]. Those results differ from those reported in our study, which found a significant association between the number of gun laws and fewer overall suicides. These results may suggest that, in the presence of gun laws, older adults are finding other, less lethal means to complete suicide. Disrupting access to firearms may only be a useful part—but only a part—of a larger, more comprehensive approach to suicide prevention.

The protective association in firearm-related suicide was stronger when focusing on gun violence restraining orders (GVRO) laws that allow for seizure of weapons from people who exhibit dangerous behavior, revealing a 2.4% reduction in firearm-related suicides among older adults in states with GVRO laws compared to states without these laws. GVRO laws were not associated with non-firearm related suicides, which is as expected given the laws’ purview. We identified two prior studies that have looked at the impact of GVRO laws on the general population. Swanson et al. (2017), in their focused study of Connecticut’s GVRO law, found that for every 10 to 20 gun seizures, one suicide was prevented [20]. Another study by Kivisto and Phalen (2018) similarly found a reduction in suicides among the general population in Connecticut and Indiana, with a 14% and 7.5% reduction in firearm suicides respectively, in the 10 years following their enactment [21]. In this latter study, however, they found that whereas Indiana demonstrated an aggregate decrease in suicide, Connecticut’s estimated reduction in firearm suicides was offset by increased non-firearm suicides. State differences in suicide rate may be contributing to these observed differences, as the overall rate of suicide in Indiana is higher than in Connecticut [33]. Across the country, overall suicide rates vary up to fourfold, from 6.9 (District of Columbia) to 29.2 (Montana) per 100,000 persons per year [33]. Differences in regional cultures might also be important to suicidality—such as in the Mountain West, dubbed the “suicide belt” due to increased mortality from suicide, where there is a pervasive cultural narrative around self-reliance and stigma about mental illness, alongside social factors such as substance use and poor economic conditions [34]. The results from this national study suggest that the net association of GVRO laws is with fewer overall suicides.

A recent study on state-level firearm ownership and suicides found that, among men, higher firearm ownership was associated with an increase in total and firearm suicide rates, and a decrease in non-firearm suicide rate, suggesting that completion of suicides may depend on access to lethal means like firearms [9]. Our data may provide additional evidence for such a trend as we found a negative association between total number of firearm laws and non-firearm related suicide rates among older adults. In fact, for both total firearm laws and for GVRO laws specifically, there was a net negative effect on suicide by any means. This is likely due to the predominant role that the availability of firearms as lethal and commonly used means plays in determining suicide rates in the United States.

Further, although our study does not differentiate between the impact on those with and without dementias—those with dementias being at higher risk of suicide due to symptoms of depression, impulsiveness, aggressiveness—the findings support a protective association of GVRO laws and total firearm laws to firearm-related suicide among the age groups most affected by a spectrum of dementia diagnoses. Future studies could elucidate the particular impact of these policies on dementia patients, including potential unintended consequences (e.g., delaying diagnosis or evaluation of symptoms out of concern for having firearm(s) confiscated; harmful downstream effects of involvement with law enforcement and the criminal justice system).

Importantly, policy level interventions should be complemented by patient-clinician level interventions, which involve increasing physician comfort with talking about guns in the home with patients and their families [35]. In one qualitative study of stakeholders at Veterans Affairs (VA) regarding mental health and suicide risk at the VA, nearly all patients felt that clinicians should routinely speak about guns with their patients, even though these conversations rarely took place [36]. Conversations should not only include discussion about whether or not to remove a weapon entirely, but also how to safeguard the home and environment, or at least restricting gun access with supervision by another family member or caregiver [35]. Just as clinicians have incorporated conversations about driving with older adults, it is critical to engage in the difficult conversation about gun ownership and gun safety. For those with dementia, discussions earlier in the course of disease is important as they may be at increased risk of attempting suicide and less likely to be supervised than later in the course of the disease [10, 37]. Conversations about firearm safety at home is also important as people may not be firearm owners themselves but be living in a household with one. At the individual level, clinicians must also be vigilant about addressing mental health symptoms and/or adjustment to cognitive decline. In one study analyzing coroner/medical examiner and law enforcement reports and suicide notes, despondency from cognitive functional decline due to dementia was believed to be a precipitant of suicide among older adults, particularly among those age 85 and older [38].

Other efforts can focus on other subgroups of older adults vulnerable to suicide—for example, older men are both more likely to own firearms and more likely to complete suicide using lethal means [39]. In the analysis of Connecticut’s GVRO law, 92% of gun removal subjects were male and 81% were cohabiting or married [20]. Therefore, even at the policy level, legislative efforts at reducing firearm ownership may or may not reduce suicide rates differently among males and females. In one national study of firearm ownership and suicide rates, there was a strong relationship between firearm ownership and suicides by any means among male, but not female, individuals [9], suggesting that policies reducing firearm ownership would reduce both total suicide rates and firearm-related suicides for males, but only firearm-related suicides for females.

This study should be viewed in light of several potential limitations. First, we cannot establish a causal relationship as this is an associative study between the firearm legislation and firearm-related suicides. However, the study was conducted over a 5-year period and we believe this adds to the robustness of our findings. Second, although we assessed the presence or absence of certain firearm legislation, we were unable to assess the effectiveness of variation amongst these laws or the effectiveness of their enforcement. That is, wide variation in policies across states includes differences in statutory requirement of the number of officers required as co-affiants, number of officers required to present before a judge, and variations in the probation period during which guns can be confiscated or an appeal process can occur [20]. These differences may also influence enforcement, similar to how high-profile events may increase awareness of GVRO laws and instigation of firearm removal from an individual deemed dangerous to themselves or others. From 1999 when the Connecticut GVRO was first passed to 2006, there were only approximately twenty guns confiscated per year; this saw a dramatic rise in 2006 after high profile mass shooting events [20]. In this light, we may even be underestimating the impact of these laws as there is often a lag in effect and increased enforcement over time. Further, given the small number of states that have either type of GVRO law currently in place, we could not differentiate between the effects of GVRO laws that grant family members versus only law enforcement officers the authority to remove firearms from an individual. As GVROs gain traction in legislative chambers, these variations will require further study as it will be vital for state and local governments to have a model policy. There is also potential for non-fatal firearm-related injury that is not captured in this study but would paint a more holistic picture of firearms’ impact on older adults and GVRO’s impact in mitigating these harms. Lastly, our analysis did not elucidate the connection between the subset of older adults with dementia diagnoses, firearm access, and suicidal behavior; future investigation in this population is warranted.

## Conclusions

We found that states with stricter firearm legislation had lower rates of firearm-relate suicides among older adults. GVRO laws were similarly protective against firearm-related suicides among older adults. GVRO legislation across the country are taking on renewed significance since recent mass shooting tragedies and our study demonstrates support for this particular firearm legislation in saving lives among older adults. We encourage future studies to confirm the positive associations we observed since the GVRO law landscape has rapidly evolved and various states have enacted GVRO laws since 2016. As GVRO legislation is introduced, researchers can also consider quasi-experimental study designs to better establish causality between GVRO laws and suicide rates. More robust research related to the impact of firearm legislation on firearm-related death in older adults is needed, alongside the implementation of evidence-based policies to reduce firearm-related suicide.

## Supplementary information

**Additional file 1 **Table 1**.** Relationship Between Total Number of Firearm Laws and Total Suicide Rate Among Older Adults from 2012 to 2016 in the US. Table 2**.** Relationship between GVRO Laws and Total Suicide Rate Among Older Adults from 2012 to 2016 in the US.

## Data Availability

The data used in the current study are present in the following publicly. available sources: the Centers for Disease Control Web-based Injury Statistics Query and Reporting System (WISQARS); the Kaiser Family Foundation; and the State Firearm Laws Database.

## Abbreviations

GVRO: Gun violence restricting order
QISQARS: Web-based injury statistics query and reporting system
