# Supplementary material for: The impact of gun violence restraining order laws in the U.S. and firearm suicide among older adults: a longitudinal state-level analysis, 2012–2016
Source: BMC Public Health. 2020 Apr 7;20:334. doi: 10.1186/s12889-020-08462-6 (PMC7137454; doi:10.1186/s12889-020-08462-6)
Supplement: Supplementary file 1 — Additional file 1 Table 1. Relationship Between Total Number of Firearm Laws and Total Suicide Rate Among Older Adults from 2012 to 2016 in the US. Table 2. Relationship between GVRO Laws and Total Suicide Rate Among Older Adults from 2012 to 2016 in the US. [file 12889_2020_8462_MOESM1_ESM.docx]

**Appendix 1**

Saadi A, Choi KR, Takada S, Zimmerman FJ. Gun Violence Restraining Order Laws in the U.S. and Firearm Suicide among Older Adults: A Longitudinal State-Level Analysis, 2012-2016

| **Table 1.** Relationship Between Total Number of Firearm Laws and Total Suicide Rate Among Older Adults from 2012-2016 in the US | |
| --- | --- |
| Elderly Population (>65 years) | Total suicide rate |
|  | Model 1 (R^2^= 0.54) |
|  | β(SE) |
| Number of firearm laws | -0.06 (0.01)** |
| Poverty rate | 0.45(0.07)** |
| Population density | -<.01(<.01)* |
| Medicaid generosity | 0.25(0.61) |
| Elderly population share | 21.10(16.40) |
| Female population share | -295.9(46.10)** |
|  |  |
| Older Adults (55–64 years) | Total suicide rate |
|  | Model 2 (R^2^= 0.51) |
|  | β(SE) |
| Number of firearm laws | -0.07(0.01)** |
| Poverty rate | 0.29(0.06)** |
| Population density | -<.01(<.01)* |
| Medicaid generosity | 0.43(0.55) |
| Older adult population share | -14.90(22.80) |
| Female population share | -272.90(36.84)** |

**Footnotes**. SE= standard error; N= 242. This table displays fixed effects models of state-level sociodemographic and policy factors predicting total suicide rate (all causes) among elderly and older adult populations in the US, excluding the District of Columbia and US territories. Models are adjusted for year. *Value is significant at the 0.05 level. **Value is significant at the 0.01 level.

| **Table 2.** Relationship between GVRO Laws and Total Suicide Rate Among Older Adults from 2012-2016 in the US | |
| --- | --- |
| Elderly Population (>65 years) | Total suicide rate |
|  | Model 3 (R^2^= 0.50) |
|  | β(SE) |
| GVRO law | -2.55(1.10)* |
| Poverty rate | 0.53(0.07)** |
| Population density | -<.01(<.01)** |
| Medicaid generosity | -1.00(0.56) |
| Elderly population share | 30.52(16.97) |
| Female population share | -355.00(46.29)** |
|  |  |
| Older Adults (55–64 years) | Total suicide rate |
|  | Model 4 (R^2^= 0.45) |
|  | β(SE) |
| GVRO law | -2.49(1.12)* |
| Poverty rate | 0.39(0.06)** |
| Population density | -<.01(<.01)** |
| Medicaid generosity | -0.98(0.52) |
| Older adult population share | 18.96(23.25) |
| Female population share | -337.30(37.02)** |

**Footnotes**. GVRO= gun violence restraining order; SE= standard error; N= 242. This table displays fixed effects models of state-level sociodemographic and policy factors predicting total suicide rate (all causes) among elderly and older adult populations in the US, excluding the District of Columbia and US territories. Models are adjusted for year. *Value is significant at the 0.05 level. **Value is significant at the 0.01 level.
